# Effect of a Brief Mindfulness-Based Program on Stress in Health Care Professionals at a US Biomedical Research Hospital: A Randomized Clinical Trial

**DOI:** 10.1001/jamanetworkopen.2020.13424

**Published:** 2020-08-25

**Authors:** Rezvan Ameli, Ninet Sinaii, Colin P. West, María José Luna, Samin Panahi, Michael Zoosman, Heather L. Rusch, Ann Berger

**Affiliations:** 1 National Institute of Mental Health, National Institutes of Health, Bethesda, Maryland; 2 Clinical Center, National Institutes of Health, Bethesda, Maryland; 3 Division of General Internal Medicine, Department of Medicine, Mayo Clinic, Rochester, Minnesota; 4 Division of Biomedical Statistics and Informatics, Department of Health Sciences Research, Mayo Clinic, Rochester, Minnesota; 5 Feinberg School of Medicine, Northwestern University, Chicago, Illinois; 6 National Institute of Nursing Research, National Institutes of Health, Bethesda, Maryland; 7 Division of Psychology, Department of Clinical Neuroscience, Karolinska Institutet, Stockholm, Sweden

## Abstract

**Importance:**

Stress among health care professionals is well documented. The use of mindfulness-based interventions to reduce stress has shown promising results; however, the time commitment of typical programs can be a barrier to successful implementation in health care settings.

**Objective:**

To determine the efficacy and feasibility of a brief mindfulness-based program to reduce stress during work hours among health care professionals.

**Design, Setting, and Participants:**

This intent-to-treat randomized clinical trial was conducted among full-time health care professionals at the Clinical Center at the National Institutes of Health in Bethesda, Maryland, between September 2017 and May 2018. Participants were randomized to receive mindfulness-based self-care (MBSC) training or life-as-usual control. Data were analyzed from June 2018 to January 2020.

**Interventions:**

The MBSC intervention included 5 weekly, 1.5-hour in-class mindfulness practice sessions.

**Main Outcomes and Measures:**

Stress level was the primary outcome, assessed with the Perceived Stress Scale 10-Item version. Secondary outcomes included anxiety, burnout, positive and negative affect, mindfulness (trait and state), and self-care. Assessments were taken at baseline and at the end of the intervention (week 5) in the intervention and control groups, and at follow-up (week 13) in the intervention group to test for a maintenance effect. A postprogram evaluation was also obtained.

**Results:**

Of 82 randomized participants, 78 who completed the study at week 5 were included in the modified intent-to-treat analysis (median [interquartile range] age, 32 [23-48] years; 65 [83%] women), including 43 participants in the MBSC group and 35 participants in the control group. At the end of the intervention, compared with the control group, the MBSC group had reduced levels of stress (mean [SD] score, 17.29 [5.84] vs 18.54 [6.30]; *P* = .02) and anxiety (mean [SD] score, 2.58 [1.52] vs 4.23 [1.73]; *P* < .001), and improved positive affect (mean [SD] score, 35.69 [7.12] vs 31.42 [7.27]; *P* < .001), state mindfulness (mean [SD] score, 3.74 [1.18] vs 2.78 [1.16]; *P* < .001), and mindful self-care (mean [SD] score, 7.29 [2.44] vs 5.54 [2.77]; *P* < .001). Burnout, negative affect, and trait mindfulness levels did not differ between groups. Changes within the MBSC group through follow-up included sustained reductions in stress (change, –6.14; 95% CI, –7.84 to –4.44; *P* < .001), anxiety (change, –1.46; 95% CI, –1.97 to –0.94; *P* < .001), trait mindfulness (change, 0.63; 95% CI, 0.36 to 0.90; *P* < .001), and state mindfulness (change, 1.89; 95% CI, 1.39 to 2.39; *P* < .001).

**Conclusions and Relevance:**

This randomized clinical trial found that this brief mindfulness-based intervention was an effective and feasible means to reduce stress in health care professionals. Larger studies are needed to assess the effects on clinical care and patient outcomes.

**Trial Registration:**

ClinicalTrials.gov Identifier: NCT03781336

## Key Points

**Question:**

Is a brief mindfulness-based program effective and feasible in reducing stress among health care professionals during work hours?

**Findings:**

In this randomized clinical trial including 78 participants randomized to a 5-session (7.5-hour total) mindfulness program or a life-as-usual control, participants in the mindfulness program reported reduced stress and anxiety compared with life-as-usual controls at the end of the intervention.

**Meaning:**

This randomized clinical trial found that this brief mindfulness intervention was an effective way of reducing stress in a health care setting.

## Teaser

This randomized clinical trial examines the effect of a brief mindfulness-based program on stress reduction among health care professionals in a US research hospital.

## Introduction

Health care professionals face challenges that can be detrimental to their physical and mental health.^1,2,3^ Untreated stress in this population can lead to anxiety, depression, substance use, sleep disorders, disrupted personal relationships, reckless behaviors, stress-related health problems, and even suicide.^1,4,5,6^ Prolonged stress can lead to burnout, a syndrome marked by symptoms of emotional exhaustion, depersonalization, and a sense of low personal accomplishment.^7,8^ Stress and burnout in health care professionals can result in decreases in job performance, job satisfaction, patient satisfaction, and quality of patient care (eg, medical errors).^2,3,9,10,11^ To help ensure the safety of both clinicians and patients, effective and feasible interventions are needed at individual and organizational levels.^3,12^

Mindfulness is defined as paying attention on purpose, in the present moment, and nonjudgmentally to the unfolding of moment-by-moment experiences.^13^ At the individual level, mindfulness-based interventions, among other benefits, have gained recognition as effective methods for reducing stress, anxiety, and burnout.^14,15^ At the organizational level, studies have shown that mindfulness increases patient satisfaction, error recognition, and clinical insight, as well as decreases medical errors.^16,17,18,19^ Recent systematic reviews and meta-analyses report overall benefits of mindfulness for health care professionals, but also note that additional well-controlled studies are needed.^20,21,22^

An important challenge to broad implementation of mindfulness programs in health care settings is the time required for training and practice. A typical mindfulness-based stress reduction (MBSR) program includes 8 weekly, 2.5- to 3-hour in-class practice sessions, 1 full-day silent retreat, and a recommendation for 45 minutes of daily practice.^13,23^ Attrition owing to time and schedule requirements and the cost of a long programs can limit their utilization at the individual and organizational levels.^24,25,26^ Abbreviated interventions using components of mindfulness have been introduced to reduce the length of programs.^26,27,28,29^ Continued studies are needed to refine programs in health care settings to maximize employee and organizational gains. The aim of this study was to assess the efficacy and feasibility of a brief mindfulness-based self-care (MBSC) program during work hours to reduce stress among health care professionals in a large clinical research setting.

## Methods

The National Institutes of Health (NIH) Office of Human Subject Research Protection approved this study and deemed it exempt from internal review board review per NIH policy (Trial Protocol in Supplement 1). Verbal consent was obtained from each participant prior to study procedures. Written consent was waived by the Office of Human Subject Research Protection owing to the deidentified nature of data collection. This single-site, parallel-group, randomized clinical trial was conducted in accordance with the Consolidated Standards of Reporting Trials (CONSORT) reporting guideline.

### Study Design, Setting, and Participants

Participants were recruited through group emails and flyers posted at the NIH Clinical Center, in Bethesda, Maryland. The study was conducted between September 2017 and May 2018 at the NIH Clinical Center, a biomedical research hospital. Participation was open to all NIH employees, contractors, and trainees. Persons with medical and psychiatric conditions were advised to consult with their health care practitioners prior to enrollment.

Participants were randomized before the start of the trial into 1 of 2 conditions, the mindfulness-based self-care (MBSC) group or the life-as-usual control group. There were 15 participants per block. This process was repeated 3 times without interaction with the applicants before group assignment. Allocated participants were then sent a coded questionnaire packet, including an informed consent describing the study. The number corresponding to each packet would link the participant to their group assignment; no personal identifiers were collected. Questionnaires were completed at baseline and immediately after the intervention (at week 5) for both groups, as well as at follow-up (at week 13) in the MBSC group to test for a maintenance effect.

### Study Groups and Intervention

A total of 82 individuals enrolled in the study; 45 participants were allocated to the MBSC group and 37 participants were allocated to the life-as-usual control group. Two participants in the MBSC group and 2 participants in the control group declined to participate after allocation, before the start of the study, and did not provide baseline data. Thus, modified intent-to-treat analyses included 43 MBSC participants and 35 controls (Figure).

**Figure.**
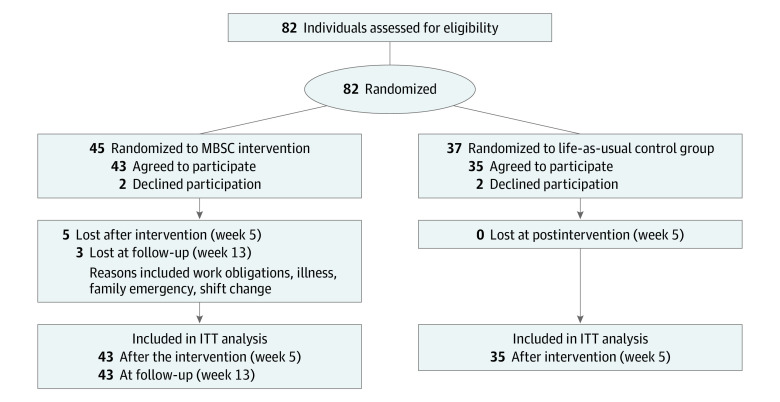
Participant Flow Diagram

Participants in the intervention group attended the MBSC, a 5-session, 7.5-hour program that was offered to health care professionals at the NIH main campus during work hours. A professionally trained teacher (R.A.) with more than 15 years of mindfulness and yoga practice experience designed and delivered the formalized program curriculum. All participants in the MBSC group received a course binder with mindfulness practice descriptions, weekly at-home practice plans, and a list of mindfulness resources. Mindfulness exercises included mindful breathing, body scan, mindful walking, mindful movements, mindful eating, and loving-kindness meditation.^13,30,31^ Participants engaged in 60 to 70 minutes of mindful practice in each class. The teacher delivered didactic material and facilitated inquiry and group discussions. Daily at-home mindfulness practice was strongly encouraged. Participants had access to reading material on mindfulness and guided meditations to facilitate at-home practice. A buddy system was established within the MBSC group to enhance a sense of community and encourage at-home practice. The in-class session themes were (1) introduction to mindfulness, (2) enhancing awareness and focused attention, (3) awareness of pleasant and unpleasant experiences, (4) transformation of difficult emotions through mindfulness, and (5) compassion. The life-as-usual control group received no instructional materials.

### Study Outcomes

The Perceived Stress Scale 10-Item version (PSS-10) was used to assess stress as the primary outcome. The PSS-10 is valid and reliable, and it is the most commonly used version of the PSS.^32,33,34^ Items are scored from 0 (indicating never) to 4 (indicating very often) with a total score ranging from 0 to 40, with higher scores indicating more stress. No cutoff scores have been established. Normative information with large samples in the US has been reported, with mean (SD) scores of 11.09 (6.77) in low-stress and 19.11 (7.92) in high-stress populations in 2009.^35,36^ Several secondary outcomes were also included. The Visual Analog Scale-Anxiety (VAS-A) was used to assess anxiety. It is a 1-item instrument, with a total possible score range from 1 to 10, with higher scores indicating more anxiety.^37^ Two items from the Maslach Burnout Inventory (MBI-2) were used^8,38^: one item assessed emotional exhaustion and a second item assessed depersonalization. These 2 items have demonstrated high factor loadings, validity, and reliability relative to the full MBI-22.^39,40^ Each of these items were separately scored on a Likert scale ranging from 0 to 6 with higher scores indicating more severity. The Positive and Negative Affect Schedule (PANAS)^41^ was used to assess positive and negative affect. These schedules each have 10 items and are rated on a 5-point Likert scale from 1 (indicating very slightly) to 5 (indicating extremely), for a total scale range of 10 to 50. The Mindful Attention Awareness Scale Trait (MAAS-T) and State (MAAS-S) were used to assess trait and state mindfulness.^42^ The MAAS-T is a 15-item instrument, with a total range from 1 to 6, with higher scores indicating higher trait mindfulness. The mean normative MAAS-T (trait mindfulness) score is 3.85, with an increase to 4.38 among Zen meditators. The MAAS-S was used to assess present moment mindfulness. It is a 5-item instrument, with a total possible range from 0 to 6, with higher scores indicating higher state mindfulness. The Mindful Self-care Scale-General (MSCS-G) was used to assess global and general self-care, including mindfulness and meditation practice.^43^ It is a 3-item inventory, scored on a scale of 0 to 4, with a total range from 0 to 12 and higher scores indicating more mindful self-care.

Feasibility was collectively assessed by response rate, attendance rate, postprogram evaluations, and reports of adverse events. The course was advertised 6 weeks prior to the first session. Response rate was defined by the course registration rate 1 week prior to the first session, which was a proxy for community interest. Completing the program (successful attendance) was defined as attending at least 3 of 5 in-class mindfulness sessions by at least 80% of the participants. A postprogram evaluation assessed various components of the program on a Likert scale from 1 (indicating poor or not helpful) to 5 (indicating excellent or very helpful). These components were considered to be of value if most participants rated them 4 or better. Adverse events were assessed by weekly class check-ins, encouraging participants to contact the instructor in case of difficulties or questions, and response to open questions in the postprogram evaluation.

### Statistical Analysis

Sample size was determined using a test for 2 means in a repeated measures design (PASS 12; NCSS) between MBSC and control groups from baseline to the end of the intervention to detect a 1.0-point difference in the primary outcome measure, PSS-10 scores. Estimates assumed a moderate-to-strong correlation (ρ = 0.5) in the repeated measures, equal group allocation, 2-sided α = .05, β = 0.90, and a 10% to 15% dropout rate, which yielded 33 participants in each group. Analyses were intent-to-treat based on randomization. Data were assessed for distributional assumptions, and either approximate normality was confirmed, or nonparametric tests were used. Categorical data (ie, demographic and clinical characteristics) were compared between MBSC and control groups using χ^2^ or Fisher exact tests, as appropriate. Continuous data were compared between groups using 2-sample *t* tests or nonparametric Wilcoxon rank sum tests. Generalized linear mixed modeling for repeated measures compared postintervention and follow-up measures between and within MBSC and control groups, as applicable. Mixed models account for correlation between repeated measurements on the same participant and accommodate missing data.^44^ Post hoc pairwise comparisons were adjusted for multiple comparisons using the Bonferroni method, and reported *P* values were corrected for multiplicity. Data are reported as frequency (percentage) and mean (SD) or median (interquartile range [IQR]). Changes between intervals and effect sizes are reported with corresponding 95% CIs. Effect size was bias-corrected Cohen *d* (Hedges *g*). All data were analyzed using SAS statistical software version 9.4 (SAS Institute). Qualitative databased on postprogram evaluations are described. Data were analyzed from June 2018 to January 2020.

## Results

### Demographic Data

Of 82 randomized participants, 78 who completed the study at week 5 were included in the modified intent-to-treat analysis (median [IQR] age, 32.0 [23.0-48.0] years; 65 [83%] women), including 43 participants in the MBSC group and 35 participants in the control group. Baseline demographic and clinical characteristics did not differ between groups (Table 1). No adverse events were reported to the teacher.

**Table 1.** Baseline Demographic and Clinical Characteristics of Study Participants

Variable	No. (%)	
	MBSC (n = 43)	Control (n = 35)
Age, median (IQR)	28.0 (23.0-49.0)	34.0 (24.0-48.0)
Women	37 (86.1)	28 (80.0)
Race/ethnicity		
American Indian or Alaska Native	1 (2.3)	0
Asian	7 (16.3)	6 (17.1)
Black	3 (7.0)	2 (5.7)
White	27 (62.8)	21 (60.0)
Mixed or other	5 (11.6)	6 (17.1)
Hispanic/Latinx	5 (11.9)	5 (14.3)
Marital status		
Single	28 (65.1)	19 (54.3)
Married	8 (18.6)	12 (34.3)
Divorced or separated	3 (7.0)	3 (8.6)
Widowed or other	4 (9.3)	1 (2.9
Religion		
Agnostic	1 (2.3)	2 (5.7)
Atheism	5 (11.6)	3 (8.6)
Buddhism	2 (4.7)	2 (5.7)
Christianity	18 (41.9)	13 (37.1)
Hinduism	3 (7.0)	2 (5.7)
Islam	2 (4.7)	3 (8.6)
Judaism	2 (4.7)	2 (5.7)
Not affiliated or other	10 (23.3)	8 (22.9)
Current position		
Administrator	7 (16.3)	3 (8.6)
Nurse	2 (4.7)	3 (8.6)
PhD scientist	6 (14.0)	3 (8.6)
Physician	7 (16.3)	11 (31.4)
Social worker	2 (4.7)	3 (8.6)
Training fellow	19 (44.2)	12 (34.3)
Education level		
Some college	1 (2.3)	1 (2.9)
Completed college	22 (51.2)	14 (40.0)
Graduate school or advanced degree	20 (46.5)	20 (57.1)
Preexisting condition		
Medical	16 (37.2)	9 (25.7)
Psychiatric	16 (38.1)	14 (40.0)

Abbreviation: MBSC, mindfulness-based self-care.

### Postintervention Assessment

At the postintervention assessment, the MBSC group, compared with the control group, had reduced levels of stress (mean [SD] PSS-10 score, 17.29 [5.84] vs 18.54 [6.30]; *P* = .02) and anxiety (mean [SD] VAS-A score, 2.58 [1.52] vs 4.23 [1.73]; *P* < .001) and improved positive affect (mean [SD] PANAS score, 35.69 [7.12] vs 31.42 [7.27]; *P* < .001), state mindfulness (mean [SD] MAAS-S score, 3.74 [1.18] vs 2.78 [1.16]; *P* < .001), and mindful self-care (mean [SD] MSCS-G score, 7.29 [2.44] vs 5.54 [2.77]; *P* < .001) (Table 2). There was no change in emotional exhaustion, depersonalization, negative affect, or trait mindfulness (Table 2).

**Table 2.** Intent-to-Treat Analysis Results for Primary and Secondary Outcomes in MBSC Participants and Life-as-Usual Controls

Outcome	Life-as-usual controls (n = 35)					Mindfulness-based self-care (n = 43)											After intervention between-group effect size (95% CI)^a^	*P* value^b^
	Score, mean (SD)		Change from baseline, mean (95% CI)	Within-group effect size (95% CI)^a^	*P* value^b^	Score, mean (SD)		Change from baseline, mean (95% CI)	Within-group effect size (95% CI)^a^	*P* value^b^	Follow-up							
	Baseline	After intervention				Baseline	After intervention				Score, mean (SD)		Change from baseline, mean (95% CI)	Within group effect size (95% CI)^a^		*P* value^b^		
Stress^c^	18.80 (6.36)	18.54 (6.30)	–0.26 (–1.88 to 1.36)	–0.04 (–0.37 to 0.29)	.99	19.63 (6.26)	17.29 (5.84)	–2.50 (–4.28 to –0.72)	–0.38 (–0.71 to –0.05)	.009	13.80 (6.45)	–6.14 (–7.84 to –4.44)			–0.90 (–1.29 to –0.50)	<.001	–0.20 (–0.66 to 0.26)	.02
Anxiety^d^	4.57 (1.69)	4.23 (1.73)	–0.34 (–0.99 to 0.30)	–0.19 (–0.53 to 0.14)	>.99	4.72 (1.62)	2.58 (1.52)	–2.13 (–2.79 to –1.48)	–1.33 (–1.78 to –0.89)	<.001	3.29 (1.47)	–1.46 (–1.97 to –0.94)			–0.90 (–1.30 to –0.51)	<.001	–1.01 (–1.49 to –0.52)	<.001
Burnout																		
Emotional exhaustion^e^	3.29 (1.45)	2.74 (1.62)	–0.54 (–1.00 to –0.08)	–0.35 (–0.69 to –0.01)	.07	2.95 (1.56)	2.89 (1.63)	–0.05 (–0.49 to 0.38)	–0.04 (–0.36 to 0.29)	>.99	2.31 (1.64)	–0.51 (–0.97 to –0.06)			–0.39 (–0.74 to –0.05)	.05	0.09 (–0.37 to 0.55)	>.99
Depersonalization^f^	1.60 (1.42)	1.57 (1.36)	–0.03 (–0.38 to 0.32)	–0.02 (–0.35 to 0.31)	>.99	1.76 (1.32)	1.22 (1.13)	–0.49 (–0.85 to –0.12)	–0.43 (–0.77 to –0.09)	.04	1.34 (1.21)	–0.18 (–0.58 to 0.23)			–0.32 (–0.66 to 0.02)	>.99	–0.28 (–0.74 to 0.19)	.47
Affect^g^																		
Positive	33.67 (5.95)	31.42 (7.27)	–2.31 (–4.35 to –0.28)	–0.33 (–0.68 to 0.02)	.18	32.85 (7.73)	35.69 (7.12)	2.94 (0.70 to 5.18)	0.37 (0.03 to 0.72)	.03	36.13 (8.43)	0.40 (0.04 to 0.76)			–0.12 (–0.57 to 0.34)	>.99	0.59 (0.10 to 1.07)	<.001
Negative	21.21 (7.27)	19.09 (7.60)	–2.13 (–4.86 to 0.61)	–0.28 (–0.63 to 0.07)	.54	21.44 (7.38)	20.73 (6.24)	–0.25 (–3.00 to 2.50)	–0.10 (–0.44 to 0.24)	>.99	18.22 (6.01)	–0.47 (–0.83 to –0.10)			–0.03 (–0.43 to 0.49)	>.99	0.23 (–0.25 to 0.72)	>.99
Mindfulness^h^																		
Trait	3.61 (0.89)	3.70 (0.96)	0.02 (–0.12 to 0.17)	0.10 (–0.24 to 0.43)	>.99	3.74 (0.99)	3.95 (0.83)	0.23 (–0.06 to 0.52)	0.23 (–0.10 to 0.55)	.18	4.33 (0.85)	0.63 (0.36 to 0.90)			0.62 (0.25 to 1.00)	<.001	0.28 (–0.19 to 0.74)	.39
State	2.48 (1.08)	2.78 (1.16)	0.25 (–0.17 to 0.67)	0.26 (–0.08 to 0.60)	>.99	2.23 (1.24)	3.74 (1.18)	1.59 (1.17 to 2.01)	1.22 (0.80 to 1.65)	<.001	4.08 (1.04)	1.89 (1.39 to 2.39)			1.46 (0.97 to 1.95)	<.001	0.81 (0.33 to 1.29)	<.001
Self-care^i^	6.00 (2.88)	5.54 (2.77)	–0.46 (–1.01 to 0.10)	–0.16 (–0.49 to 0.17)	>.99	5.60 (2.75)	7.29 (2.44)	1.61 (0.68 to 2.53)	0.64 (0.29 to 0.99)	<.001	6.37 (2.76)	0.40 (–0.68 to 1.48)			0.27 (–0.06 to 0.61)	.85	0.67 (0.19 to 1.14)	<.001

^a^ Effect sizes are bias-corrected Cohen *d* (Hedges *g*), in which 0.2 = small effect, 0.5 = medium effect, and 0.8 = large effect. Computations were based on subtracting control data from intervention data, and former intervals subtracted from later intervals; thus, a positive effect size indicates an increase in the mean due to the effect of the intervention, and a negative effect size indicates a decrease in the mean due to the effect of the intervention. Mean change may not equal the difference in the means if data were missing at either interval.

^b^ *P* values are corrected for multiple comparisons.

^c^ Measured using the Perceived Stress Scale 10-Item version.

^d^ Measured using the Visual Analog Scale–Anxiety.

^e^ Measured using Maslach Burnout Inventory Item 1.

^f^ Measured using Maslach Burnout Inventory Item 2.

^g^ Measured using Positive and Negative Affect Schedule.

^h^ Measured using Mindful Attention Awareness Scales for trait and state.

^i^ Measured using Mindful Self-Care Scale–General.

Among MBSC participants, stress was reduced at the end of the intervention compared with baseline (change, –2.50; 95% CI, –4.28 to –0.72; *P* = .009). In addition, anxiety (change, –2.13; 95% CI, –2.79 to –1.48; *P* < .001), depersonalization (change, –0.49; 95% CI, –0.85 to –0.12, *P* = .04), positive affect (change, 2.94; 95% CI, 0.70 to 5.18; *P* = .027), state mindfulness (change, 1.59; 95% CI, 1.17 to 2.01; *P* < .001), and mindful self-care (change, 1.61; 95% CI, 0.68 to 2.53; *P* < .001) were also improved from baseline to the end of the intervention (Table 2). There was no change in emotional exhaustion, negative affect, and trait mindfulness from baseline to after the intervention among MBSC participants. The control group showed no changes in any of the primary or secondary outcomes during this interval.

### Follow-up

From the end of the intervention to the 13-week follow-up, there was a maintenance effect for stress (change, –6.14; 95% CI, –7.84 to –4.44; *P* < .001), anxiety (change, –1.46; 95% CI, –1.97 to –0.94; *P* < .001), trait mindfulness (change, 0.63; 95% CI, 0.36 to 0.90; *P* < .001), and state mindfulness (change, 1.89; 95% CI, 1.39 to 2.39; *P* < .001) in the MBSC group. Burnout, positive affect, and mindful self-care gains were not maintained (Table 2).

### Feasibility

Classes filled within 2 to 3 days of announcements with waiting lists, indicating a high response rate and interest in mindfulness in the workplace. Of 43 participants, 35 completed the program (ie, attended a minimum of 3 sessions). Thus, the adherence rate was 81.4%. Participants who completed the program and participants who did not complete the program did not differ on baseline demographic or clinical characteristics. A total of 35 participants (81.4%) provided postprogram evaluations. The overall quality of the program was rated as 4 or 5 (ie, very good to excellent) by 34 participants (97.1%). Also, 32 participants (91.4%) reported that the course was very helpful and improved their overall quality of life (ratings of 4-5). Individual practices were rated 4 or 5 by 34 participants (94.3%) for mindful breathing, 34 participants (94.3%) for body scan, 28 participants (80.0%) for mindful movements, 25 participants (71.4%) mindful walking, 18 participants for (51.4%) for mindful eating, 26 participants (74.3%) for mindful attention to pleasurable experiences, 28 participants (80.0%) for mindful attention to difficult experiences, and 33 participants (94.3%) for loving kindness meditation.

## Discussion

The results of this randomized clinical trial support the effectiveness of a brief mindfulness-based program during work hours for reducing stress in a mixed group of health care professionals. The program was also effective in reducing anxiety and improving positive affect, state mindfulness, and self-care in the MBSC participants. Burnout (ie, depersonalization and emotional exhaustion), negative affect, and trait mindfulness did not differ between groups. Within-group analyses from postintervention to follow-up indicated maintained improvements in stress, anxiety, and trait and state mindfulness in the MBSC participants. However, the postintervention within-group improvements in self-care and depersonalization were not maintained at the 13-week follow-up.

Stress reduction is clearly desirable at the individual and organizational levels. Typical MBSR programs are approximately 30 hours in length, include a full-day silent retreat, and are relatively impractical and costly to implement during work hours in health care settings.^24,26^ When mindfulness-based interventions are very brief (ie, <4 hours), the benefits are inconclusive.^26,45^ The 7.5-hour MBSC program implemented in this study may represent an effective and feasible level of mindfulness-based training for busy health care professionals. It may also allow for manageable costs to the organization without losing the essence of what MBSR programs offer (ie, stress reduction). Another commonly reported effect of full-length mindfulness-based programs is reduction in anxiety.^46,47^ Our intervention was also effective in reducing anxiety among participants. Reduction in both stress and anxiety was maintained at follow-up, lending support to the current level of training as an effective alternative to a full-length mindfulness-based program.

The intervention did not have an effect on burnout between the 2 groups, and within-group improvement in depersonalization in the MBSC participants was not maintained at follow-up. The absence of an effect on burnout could have been due to a floor effect or the focused utilization of 2 items from the MBI-2. Participants in our study did not endorse high levels of emotional exhaustion or depersonalization at baseline. Future studies may consider a more comprehensive assessment of all aspects of burnout syndrome and use the MBI 22-Item in its entirety. Alternatively, burnout levels could be part of the participant inclusion criteria from the outset if burnout is the primary outcome of interest.

In this randomized clinical trial, trait and state mindfulness were assessed to evaluate the effect of the intervention on this construct. State mindfulness improved at the end of the intervention and follow-up. Trait mindfulness, which did not improve at the end of the intervention, significantly improved at follow-up. It is notable that temporal sequencing studies support the notion that change in some psychological variables may take time to manifest. Similarly, it is believed that some critical threshold of practice is needed before change can occur.^27,48^

This randomized clinical trial provides support for the MBSC program’s feasibility. The classes filled up quickly, 81% of participants completed the program, postprogram evaluations were positive, and no adverse events were reported. Compared with participants who completed the program, participants who did not complete the program did not differ on any of the baseline outcome variables or demographic characteristics; therefore, a systematic selection bias is unlikely.^49,50^ Work obligations, illness, family emergencies, and shift changes were the reasons given for nonattendance. The 19% attrition rate is consistent with the literature for similar populations. For example, attrition rates of 22.7% in health care professionals and 34.9% in mental health professionals who took a course of MBSR have been reported.^51^ Another report, a meta-analysis of mindfulness studies in nonclinical populations,^52^ reported attrition rates of 3% to 51%. In nonclinical populations, higher attendance rates are more common among college students who may have protected time and tangible incentives for attendance.^52^

### Limitations

This study has several limitations. Despite the statistical significance of these findings, we are limited in the conclusions we can infer regarding clinical significance since there are no established minimal important difference scores for the PSS-10 or other measures we used. For the PSS-10, the observed between-group effect size of 0.2 is consistent with a small effect. Normative data from 2009 indicate that in the general public, a mean difference of 8 points distinguishes low-stress populations from high-stress populations.^35^ In addition, a 0.25- to 0.50-SD change is an effect size considered to indicate meaningful change for quality of life measures.^53,54^ Scores for the PSS-10 in our MBSC participants were reduced by a mean of 2.5 points at week 5 and 6.1 points at week 13, representing 0.4- to 0.9-SD reductions.

This study was conducted in a research hospital with predominantly women participants with a high level of education. The generalizability of these results to other types of organizations, lower educational levels, and men may be limited. A 2015 meta-analysis of mindfulness studies in nonclinical populations^52^ found that 56% to 100% of participants were women with a mean of 78% across studies. Given the documented benefits of mindfulness and other complementary and alternative interventions and their contribution to health and well-being, it is important to identify factors that could assist in increasing men’s participation in these practices.^55,56,57,58^

The use of a life-as-usual vs an active control group is another limitation of this study. The inclusion of an active control group would enhance our understanding of the unique contributions of mindfulness. However, there is no consensus as to what constitutes an ideal control group for mindfulness-based programs. Yoga, tai chi, and relaxation practices, which are often used as control groups in nonclinical populations, typically incorporate elements that are also present in mindfulness practices. A 2015 meta-analysis^21^ reported that both within- and between-group effect sizes were robust with or without active control groups in studies of psychological distress. Sham mindfulness instructions during short interventions (eg, 20-30 minutes) have also demonstrated the effectiveness of mindfulness.^59^ This type of control intervention is quite interesting and creative. However, producing sham mindfulness becomes much more challenging when long programs across several sessions are considered. Further limitations of our study include possible expectancy bias in self-motivating volunteers, and the reliance on self-report measures that are prone to social desirability bias, which we attempted to mitigate through the collection of anonymized data.

## Conclusions

This randomized clinical trial found that a brief course of mindfulness training during work hours was feasible and effective in reducing stress among a mixed group of health care professionals. Engaging both individual and organizational involvement toward reducing stress and enhancing mindfulness may have far-reaching effects on employee health, patient outcomes, and organizational success. The effect of employee gains on patient outcomes remains an important subject for future inquiries.

## Notes

Supplement 1. **Trial Protocol**

Supplement 2. **Data Sharing Statement**
