# Supplementary material for: Effect of a Brief Mindfulness-Based Program on Stress in Health Care Professionals at a US Biomedical Research Hospital: A Randomized Clinical Trial
Source: JAMA Netw Open. 2020 Aug 25;3(8):e2013424. doi: 10.1001/jamanetworkopen.2020.13424 (PMC7448827; doi:10.1001/jamanetworkopen.2020.13424)
Supplement: Supplement 1. — Trial Protocol [file jamanetwopen-3-e2013424-s001.pdf]

Your Answers for OHSRP Determination '17-NIMH-00118' (05/08/2017)

You may click here to return to OHSRP's Determinations website at any time.

SHOULD I STOP OR GO FORWARD WITH AN OHSRP SUBMISSION?

Question
"If this is a new request (i.e. not an amendment to a previously approved project), have you already started or completed your research activity? (If amending your project select "No" below) "

Your Answer
• No

Question
"Are the specimens/data that will be used in this project coming from an active, NIH IRB-approved protocol; or is the proposed activity a component of an active, NIH IRB-approved protocol, (e.g. the results of this activity will be used in support of the protocol)?"

Your Answer
• No

Question
"Does the NIH investigator or NIH collaborator know the identity of (have identifiers associated with) the living human sources of the specimens/data that will be used on this project? (If the specimens/data are not individually identifiable, but the identity of the subjects may readily be ascertained by the investigator, e.g. because of a small sample size, please answer "Yes" below. Answer "No" below if interacting with subjects to conduct survey, interview or focus group research)."

Your Answer
• No

Question
"Does this activity involve prisoners?"

Your Answer
• No

Question
"For this project, is NIH conducting a research activity that is part of an FDA-regulated protocol approved by an IRB, e.g. the protocol is studying the safety or efficacy of unapproved drugs/devices or new uses of approved drugs/devices?"

Your Answer
• No

Question

"* If yes, has the collaborator confirmed that the planned research activity, which will occur at NIH, is included in the IRB/ethics committee-approved protocol and consent form at his/her institution?"

Your Answer

** DID NOT ANSWER **

SECTION I - GENERAL INFORMATION

Question
"Project Title:"

Your Answer
Mindfulness Based Self Care (MBSC)

Question
"Project Description:"

Your Answer
At the Clinical Center Pain and Palliative Care Service (PPCS) we are currently developing a self-care program for clinical fellows and attending physicians, the Mindfulness Based Self-Care (MBSC) program. This program incorporates key components of the well-known mindfulness based stress reduction (MBSR). The program will offer 5 one and a half hour sessions for a total of 7.5 hours. The program will be offered to Hospice and Palliative Medicine fellows, first, second, and third year Hematology-Oncology fellows. We plan to have 10-15 participants per session on a first come first served basis. If successful, we will offer this class multiple times throughout the year so everyone who is interested will be able to participate. See attached narrative.

Question
"Upload Additional Project Information or Narrative Template (More Information):"

Your Answer
Narrative_Template_for_Survey_Research_rev._5_23_17.docx

Question
"Proposed Start Date:"

Your Answer
07/05/2017

Question
"Proposed Completion Date:"

Your Answer
07/01/2019

Question
"Search for Requestor (your name):"

Your Answer
Rezvan Ameli

Question
"If you are not the Senior Investigator (SI), what is your role?"

Your Answer
- Other investigator

Question

"Other - please enter additional information here"

Your Answer
** DID NOT ANSWER **

Question
"Search for Senior Investigator*:"

Your Answer
Rezvan Ameli

Question
"Search for Supervisor**:"

Your Answer
Ann M Cohen-Berger

Question
"Are there any additional NIH Investigators on your team conducting this research (e.g. junior investigator, contractor, fellow, student, etc...)?"

Your Answer
- No

Question
"Search for Additional Investigator #1:"

Your Answer
** DID NOT ANSWER **

Question
"Search for Additional Investigator #2:"

Your Answer
** DID NOT ANSWER **

Question
"Search for Additional Investigator #3:"

Your Answer
** DID NOT ANSWER **

Question
"Search for Additional Investigator #4:"

Your Answer
** DID NOT ANSWER **

Question
"Search for Additional Investigator #5:"

Your Answer

** DID NOT ANSWER **

SECTION II - SPECIAL CATEGORIES

Question
"Only select the applicable activities or materials below, if they are involved in the project.   PLEASE NOTE: The majority of projects do not involve any of the activities listed below. Skip to the next question unless your are sure your activities are listed below. (Select all that apply.)"

Your Answer
** DID NOT ANSWER **

Question
"I (the Senior Investigator) certify that the proposed project:"

Your Answer
• Involves other types of specimens/data or research activities than those listed above. Please continue completing the request for determination form.

SECTION III - COLLABORATORS AND OTHER ENTITIES

Question
"Collaborator #1:"

Your Answer
** DID NOT ANSWER **

Question
"Institution/IC Name:"

Your Answer
** DID NOT ANSWER **

Question
"FWA #:"

Your Answer
** DID NOT ANSWER **

Question
"City/State/Country:"

Your Answer
** DID NOT ANSWER **

Question
"Email Address:"

Your Answer
** DID NOT ANSWER **

Question
"Collaborator Actions"

Your Answer
- N/A

Question
"Additional Collaborator Details:"

Your Answer
** DID NOT ANSWER **

Question
"Collaborator #2:"

Your Answer
** DID NOT ANSWER **

Question
"Institution/IC Name:"

Your Answer
** DID NOT ANSWER **

Question
"FWA #:"

Your Answer
** DID NOT ANSWER **

Question
"City/State/Country:"

Your Answer
** DID NOT ANSWER **

Question
"Email Address:"

Your Answer
** DID NOT ANSWER **

Question
"Collaborator Actions"

Your Answer
** DID NOT ANSWER **

Question
"Additional Collaborator Details:"

Your Answer
** DID NOT ANSWER **

Question
"Collaborator #3:"

Your Answer
** DID NOT ANSWER **

Question
"Institution/IC Name:"

Your Answer
** DID NOT ANSWER **

Question
"FWA #:"

Your Answer
** DID NOT ANSWER **

Question
"City/State/Country:"

Your Answer
** DID NOT ANSWER **

Question
"Email Address:"

Your Answer
** DID NOT ANSWER **

Question
"Collaborator Actions"

Your Answer
** DID NOT ANSWER **

Question
"Additional Collaborator Details:"

Your Answer
** DID NOT ANSWER **

Question
"Upload Additional Collaborator Information:"

Your Answer
** DID NOT ANSWER **

SECTION IV - RESEARCH WITH SPECIMENS AND DATA

Question
"Does this activity include any of the following? (Select all that apply)"

Your Answer
• Prospective data collection involving Survey, Interview/Focus Group procedures, Observation of public behavior, Educational tests, Educational research, or Research on Public benefit/service programs
• Program Evaluation (not meeting the definition under Special Categories)

Question
"Other - please enter additional information"

Your Answer
** DID NOT ANSWER **

Question
"If you selected Program Evaluation or QA/QI above, does the activity only involve interview or survey procedures?"

Your Answer
• Yes

Question
"What role(s) will the NIH Investigator(s) have on this research project? (Select all that apply)"

Your Answer
• Interacting with subjects to conduct surveys, interviews/focus groups, observations of public behavior, educational research or tests, or research on public benefit or service programs
• Analyzing specimens or data
• Authoring publication(s)/manuscript(s) pertaining to this research

---

Question
"Other - please enter additional information here"

Your Answer
** DID NOT ANSWER **

Question
"Identify the types of data or specimen involved in this project (Select all that apply)"

Your Answer
• Data, specify below:

Question
"Medical Records - specify source"

Your Answer
** DID NOT ANSWER **

Question
"Data - specify type"

Your Answer
questionnaires

Question
"Specimens - specify type"

Your Answer
** DID NOT ANSWER **

Question
"Imaging - specify type"

Your Answer
** DID NOT ANSWER **

Question
"Other - please enter additional information here"

Your Answer
** DID NOT ANSWER **

Question
"Biorepository - specify name"

Your Answer
** DID NOT ANSWER **

Question
"Database or website - Enter name and URL"

Your Answer
** DID NOT ANSWER **

Question
"Select attachment to upload:"

Your Answer
Doc1.docx

SECTION IV - RESEARCH WITH SPECIMENS AND DATA - Continued

Question
"Do all the specimens/data or information already exist?"

Your Answer
- No

Question
"If Yes, were the specimens/data for this project originally collected for:"

Your Answer
- N/A

Question
"Is/was there IRB/Ethics Committee approval for the collection or use of the specimens/data at your collaborator's site (if applicable)?"

Your Answer
- No

Question
"Please select the response that best describes the specimens/data utilized for this activity: (Please confirm this with your collaborator, if applicable, prior to submitting this form.)"

Your Answer
- Specimens/data will not be coded nor contain any identifiable information that can be used to identify the human source(s), and cannot be linked to individual subjects by you or your collaborators (if applicable).

Question
"If an existing identifiable specimens/data set will be de-identified before the research commences, please indicate who will conduct the de-identification:"

Your Answer
- N/A

Question
"NIH SENIOR INVESTIGATOR CERTIFICATION FOR RESEARCH INVOLVING CODED SPECIMENS/DATA Answer below only when the other party can identify the human source(s) directly, through a code-key or when using an Honest Broker:
I (the Senior Investigator) certify that I will not be able to re-identify the human source(s) of the specimens/data in this project, and I have obtained one or more of the following to confirm this. Further, I promise to retain this documentation and provide it upon request. (Select all that apply)"

Your Answer
** DID NOT ANSWER **

Question
"If applicable, will the recipient of the specimens/data be returning results to the sender? (Select all that apply)"

Your Answer

** DID NOT ANSWER **

Question
"* If coded results are being returned to a collaborator who has the code-key to re-identify the human sources of the specimens/data, is there IRB/ethics committee approval for this research collaboration at the collaborator's institution?"

Your Answer
** DID NOT ANSWER **

Question
"Additional Info:"

Your Answer
** DID NOT ANSWER **

SECTION V - RESEARCH INVOLVING EDUCATIONAL RESEARCH OR TESTING, SURVEY OR INTERVIEW PROCEDURES, OR OBSERVATION OF PUBLIC BEHAVIOR

Question
"Specify the types of research or procedures involved in this project: (Select all that apply)"

Your Answer
- Survey

Question
"Other - please enter additional information here"

Your Answer
** DID NOT ANSWER **

Question
"How will data collection be conducted? (Select all that apply)"

Your Answer
- In-person at an NIH site
- Other, specify below:

Question
"In-person at my collaborator's institution(s) or research site(s) - please enter additional information here"

Your Answer
** DID NOT ANSWER **

Question
"In-person at another site(s) - please enter additional information here"

Your Answer
** DID NOT ANSWER **

Question
"Other - please enter additional information here"

Your Answer
Possibly completed by participants in their homes

Question
"Who will be conducting the data collection? (Select all that apply)"

Your Answer
- Other, specify below:

Question
"Off-site contractor - please enter additional information here"

Your Answer

** DID NOT ANSWER **

Question
"Online survey tool - please enter additional information here"

Your Answer
** DID NOT ANSWER **

Question
"Other - please enter additional information here"

Your Answer
Participants will complete the surveys on paper

Question
"What is the age range of subjects involved in the research?"

Your Answer
- Adults aged >= 18 years

Question
"* If children aged less than 18 years and the project involves observation of public behavior, will the NIH investigator(s) participate in the activities being observed?"

Your Answer
** DID NOT ANSWER **

Question
"Does your project fall into any of the categories of 'clinical research' as defined by the NIH? (See NIH Glossary & Acronym List for the full NIH definition of 'clinical research'.)"

Your Answer
- NONE OF THE ABOVE

Question
"If the Planned Enrollment Report is required per above, confirm that it has been approved by the IC approver as scientifically valid: (This step must be completed before uploading)"

Your Answer
** DID NOT ANSWER **

Question
"What is the z-number for this project?"

Your Answer
** DID NOT ANSWER **

Question
"Upload Planned Enrollment Report"

Your Answer

** DID NOT ANSWER **

Question
"Upload Consent Language/Script"

Your Answer
** DID NOT ANSWER **

Question
"Upload Survey Instrument /Interview Questions /Focus Group Script"

Your Answer
Measurement-full_set_of_measures_for_the_MBSC_program_5-23-17.pdf

Question
"Additional Info:"

Your Answer
The consent information is included in the narrative template.

HRPP HUMAN RESEARCH PROTECTIONS PROGRAM
Intramural Research Program

Your Answers for OHSRP Determination '17-NIMH-00118-1' (08/25/2017)

You may click here to return to OHSRP's Determinations website at any time.

SHOULD I STOP OR GO FORWARD WITH AN OHSRP SUBMISSION?

Question
"If this is a new request (i.e. not an amendment to a previously approved project), have you already started or completed your research activity? (If amending your project select "No" below) "

Your Answer
- No

Question
"Are the specimens/data that will be used in this project coming from an active, NIH IRB-approved protocol; or is the proposed activity a component of an active, NIH IRB-approved protocol, (e.g. the results of this activity will be used in support of the protocol)?"

Your Answer
- No

Question
"Does the NIH investigator or NIH collaborator know the identity of (have identifiers associated with) the living human sources of the specimens/data that will be used on this project? (If the specimens/data are not individually identifiable, but the identity of the subjects may readily be ascertained by the investigator, e.g. because of a small sample size, please answer "Yes" below. Answer "No" below if interacting with subjects to conduct survey, interview or focus group research)."

Your Answer
- No

Question
"Does this activity involve prisoners?"

Your Answer
- No

Question
"For this project, is NIH conducting a research activity that is part of an FDA-regulated protocol approved by an IRB, e.g. the protocol is studying the safety or efficacy of unapproved drugs/devices or new uses of approved drugs/devices?"

Your Answer
- No

Question

"* If yes, has the collaborator confirmed that the planned research activity, which will occur at NIH, is included in the IRB/ethics committee-approved protocol and consent form at his/her institution?"

Your Answer

** DID NOT ANSWER **

SECTION I - GENERAL INFORMATION

Question
"Project Title:"

Your Answer
Mindfulness Based Self Care (MBSC)

Question
"Project Description:"

Your Answer
This is an amendment. See narrative for details. Below is a summary of revisions:
A. The evaluation will involve NIH staff and trainees instead of just fellows.
B. We will attempt to recruit an equal number of participants and non-participants (to act as controls) to participate in the evaluation. Controls will be chosen from the waiting list of participants who havent participated.
C. Recruitment will all be done via flyers.
D. The timing of the instruments has changed from what it was previously. We added 3 administrations to the two-item burn out measure (which we are already administering pre-post) for the participant group. The MBI (2 items) instrument will now be completed by MBSC participants before classes 2, 3, 4, and 5, along with the Stress/Anxiety Thermometer (1 item), the MASS at Time of Assessment (5 items), and the Mindful Self-Care (3 items) instruments. The Stress/Anxiety Thermometer (1 item), the MASS at Time of Assessment (5 items), and the Mindful Self-Care (3 items) instruments will be repeated after classes 1,2,3,and 4.
E. The controls (non-participants) will now fill out surveys on 3 instead of 6 occasions.
F. I have removed the Activity Record as one of the instruments that the non-participants complete.
G. The class evaluation form has been modified to reflect ratings of the various techniques used during the sessions instead of rank ordering.
H. I have modified the attached narrative template including the consent language and the narrative template to reflect these changes.

Question
"Upload Additional Project Information or Narrative Template (More Information):"

Your Answer
Narrative_Template_for_Survey_Research_9_13_17.docx

Question
"Proposed Start Date:"

Your Answer
10/01/2017

Question
"Proposed Completion Date:"

Your Answer
10/01/2020

Question
"Search for Requestor (your name):"

Your Answer
Rezvan Ameli

Question
"If you are not the Senior Investigator (SI), what is your role?"

Your Answer
- Other investigator

Question
"Other - please enter additional information here"

Your Answer
** DID NOT ANSWER **

Question
"Search for Senior Investigator*:"

Your Answer
Rezvan Ameli

Question
"Search for Supervisor**:"

Your Answer
Ann M Cohen-Berger

Question
"Are there any additional NIH Investigators on your team conducting this research (e.g. junior investigator, contractor, fellow, student, etc...)?"

Your Answer
- No

Question
"Search for Additional Investigator #1:"

Your Answer
Ninet Sinaii

Question
"Search for Additional Investigator #2:"

Your Answer
** DID NOT ANSWER **

Question
"Search for Additional Investigator #3:"

Your Answer
** DID NOT ANSWER **

Question
"Search for Additional Investigator #4:"

Your Answer
** DID NOT ANSWER **

Question
"Search for Additional Investigator #5:"

Your Answer
** DID NOT ANSWER **

SECTION II - SPECIAL CATEGORIES

Question
"Only select the applicable activities or materials below, if they are involved in the project.   PLEASE NOTE: The majority of projects do not involve any of the activities listed below. Skip to the next question unless your are sure your activities are listed below. (Select all that apply.)"

Your Answer
** DID NOT ANSWER **

Question
"I (the Senior Investigator) certify that the proposed project:"

Your Answer
• Involves other types of specimens/data or research activities than those listed above. Please continue completing the request for determination form.

SECTION III - COLLABORATORS AND OTHER ENTITIES

Question
"Collaborator #1:"

Your Answer
** DID NOT ANSWER **

Question
"Institution/IC Name:"

Your Answer
** DID NOT ANSWER **

Question
"FWA #:"

Your Answer
** DID NOT ANSWER **

Question
"City/State/Country:"

Your Answer
** DID NOT ANSWER **

Question
"Email Address:"

Your Answer
** DID NOT ANSWER **

Question
"Collaborator Actions"

Your Answer
- N/A

Question
"Additional Collaborator Details:"

Your Answer
** DID NOT ANSWER **

Question
"Collaborator #2:"

Your Answer
** DID NOT ANSWER **

Question
"Institution/IC Name:"

Your Answer
** DID NOT ANSWER **

Question
"FWA #:"

Your Answer
** DID NOT ANSWER **

Question
"City/State/Country:"

Your Answer
** DID NOT ANSWER **

Question
"Email Address:"

Your Answer
** DID NOT ANSWER **

Question
"Collaborator Actions"

Your Answer
** DID NOT ANSWER **

Question
"Additional Collaborator Details:"

Your Answer
** DID NOT ANSWER **

Question
"Collaborator #3:"

Your Answer
** DID NOT ANSWER **

Question
"Institution/IC Name:"

Your Answer
** DID NOT ANSWER **

Question
"FWA #:"

Your Answer
** DID NOT ANSWER **

Question
"City/State/Country:"

Your Answer
** DID NOT ANSWER **

Question
"Email Address:"

Your Answer
** DID NOT ANSWER **

Question
"Collaborator Actions"

Your Answer
** DID NOT ANSWER **

Question
"Additional Collaborator Details:"

Your Answer
** DID NOT ANSWER **

Question
"Upload Additional Collaborator Information:"

Your Answer
** DID NOT ANSWER **

SECTION IV - RESEARCH WITH SPECIMENS AND DATA

Question
"Does this activity include any of the following? (Select all that apply)"

Your Answer
• Prospective data collection involving Survey, Interview/Focus Group procedures, Observation of public behavior, Educational tests, Educational research, or Research on Public benefit/service programs
• Program Evaluation (not meeting the definition under Special Categories)

Question
"Other - please enter additional information"

Your Answer
** DID NOT ANSWER **

Question
"If you selected Program Evaluation or QA/QI above, does the activity only involve interview or survey procedures?"

Your Answer
• Yes

Question
"What role(s) will the NIH Investigator(s) have on this research project? (Select all that apply)"

Your Answer
• Interacting with subjects to conduct surveys, interviews/focus groups, observations of public behavior, educational research or tests, or research on public benefit or service programs
• Analyzing specimens or data
• Authoring publication(s)/manuscript(s) pertaining to this research

---

Question
"Other - please enter additional information here"

Your Answer
** DID NOT ANSWER **

Question
"Identify the types of data or specimen involved in this project (Select all that apply)"

Your Answer
• Data, specify below:

Question
"Medical Records - specify source"

Your Answer
** DID NOT ANSWER **

Question
"Data - specify type"

Your Answer
questionnaires

Question
"Specimens - specify type"

Your Answer
** DID NOT ANSWER **

Question
"Imaging - specify type"

Your Answer
** DID NOT ANSWER **

Question
"Other - please enter additional information here"

Your Answer
** DID NOT ANSWER **

Question
"Biorepository - specify name"

Your Answer
** DID NOT ANSWER **

Question
"Database or website - Enter name and URL"

Your Answer
** DID NOT ANSWER **

Question
"Select attachment to upload:"

Your Answer
Combined_measures_9_13_17.docx

SECTION IV - RESEARCH WITH SPECIMENS AND DATA - Continued

Question
"Do all the specimens/data or information already exist?"

Your Answer
• No

Question
"If Yes, were the specimens/data for this project originally collected for:"

Your Answer
• N/A

Question
"Is/was there IRB/Ethics Committee approval for the collection or use of the specimens/data at your collaborator's site (if applicable)?"

Your Answer
• No

Question
"Please select the response that best describes the specimens/data utilized for this activity: (Please confirm this with your collaborator, if applicable, prior to submitting this form.)"

Your Answer
• Specimens/data will not be coded nor contain any identifiable information that can be used to identify the human source(s), and cannot be linked to individual subjects by you or your collaborators (if applicable).

Question
"If an existing identifiable specimens/data set will be de-identified before the research commences, please indicate who will conduct the de-identification:"

Your Answer
• N/A

Question
"NIH SENIOR INVESTIGATOR CERTIFICATION FOR RESEARCH INVOLVING CODED SPECIMENS/DATA Answer below only when the other party can identify the human source(s) directly, through a code-key or when using an Honest Broker:
I (the Senior Investigator) certify that I will not be able to re-identify the human source(s) of the specimens/data in this project, and I have obtained one or more of the following to confirm this. Further, I promise to retain this documentation and provide it upon request. (Select all that apply)"

Your Answer
** DID NOT ANSWER **

Question
"If applicable, will the recipient of the specimens/data be returning results to the sender? (Select all that apply)"

Your Answer

** DID NOT ANSWER **

Question
"* If coded results are being returned to a collaborator who has the code-key to re-identify the human sources of the specimens/data, is there IRB/ethics committee approval for this research collaboration at the collaborator's institution?"

Your Answer
** DID NOT ANSWER **

Question
"Additional Info:"

Your Answer
Below is the tracked version of the Narrative :
Narrative Template for Educational, Survey or Interview Research
Prepare a narrative, either within the project description section of the form, or upload an attachment into the web-based system, which addresses all the questions below. Be sure to discuss in detail the role of the NIH research team and every other entity who will assist or collaborate in the research, including companies with whom you are subcontracting. Collaborators or subcontractors, who are not NIH employees and who are conducting the research off-site, will commonly need IRB approval or an exemption from their own IRB of record.
Remember to also upload a copy of any recruitment scripts, screening questions, survey, interview, or focus group questions or scripts as well as the consent language that will be used for your project into the system. Note that the functionality in the system only allows one document per upload. If you have more than three documents to upload, combine them into a single PDF and upload that instead.
1. What is the research question or purpose of this research activity?

At the Clinical Center Pain and Palliative Care Service (PPCS) we are currently offering a self-care program to NIH staff and trainees, developing a self-care program for clinical fellows and attending physicians, the Mindfulness Based Self-Care (MBSC) program. This program incorporates key components of the well-known mindfulness based stress reduction (MBSR) which typically spans over 9 sessions for 30 hours. In our program, 5 sessions will be devoted to this activity and each class will be 1.5 hours in length for a total of 7.5 hours. Exposure to mindfulness based practices are found to be effective even when shorter than 30 hours of teachings are provided. Initially, Wwe will conduct an evaluation of the effectiveness of the program by comparing MBSC class participants responses over time as well as comparing their responses to those of non-participants. These participants will all be NIH staff and trainees. all be clinical fellows.

2. Describe the target population, including the number of planned participants. Subject selection must be equitable or otherwise justified here. (See NIH HRPP SOP 6, 6.12.)

The program is offered to NIH staff and trainees. will be offered to Hospice and Palliative Medicine, as well as first, second, and third year Hematology-Oncology fellows. Maximum enrollment is 15 We plan to have 10-15 participants per session on a first come first served basis. If successful, wWe will offer this class multiple times throughout the year so anyone who is interested will be able to participate. Approximately 40-50 fellows could potentially partake in the program this year. Moving forward the program will also be offered to attending physicians. We will also attempt to recruit an equal number of 20-25 non-participants to serve as a control group for the project.

3. Which entity(ies) will conduct recruitment? How will it be conducted (telephone, email, regular mail, on-line, in person or some combination of these)? If one entity will simply send out a link through a mass mailing, state that. Will that entity receive and/or maintain any personal identifiers as part of this process? If so, what type of identifiers? (See the list of possible identifiers is included in NIH HRPP SOP 5, Appendix 2.)

This project doesnt involve any outside collaborators. The class will be advertised by flyers and emails that will include

relevant (dates, times, location, content) class information. an initial introductory lecture during the fellows orientation sessions and followed up by emails from NIH training directors/designee and Rezvan Ameli, Ph.D. that will include the class information, brief description of each session, and information about the program evaluation.
AAfter finalizing the list of participants, they will be invited to participate in the project as a volunteer. Those on waiting list will be invited to participate as the control subject group. fter the fellows have signed up for the class, another email will be sent out inviting those who have not signed up to participate in the evaluation portion only. Dr. Ameli will have email addresses and names as part of her role in recruiting for, teaching the classes, and implementing the evaluation, but none of the questionnaire data collected will be linked to these personal identifiers.

4. If the project involves any pre-screening for eligibility, which entity(ies) will conduct pre-screening? How will it be conducted specifically (telephone, email, regular mail, on-line, in person or some combination of these)? Will that entity receive and/or maintain any personal identifiers as part of this process? If so, what type of identifiers?

N/A.

5. What type of research procedures will be used? For example, will this project involve surveys, interviews, focus groups, educational tests, etc.? How will data collection be conducted (telephone, email, regular mail, on-line, in person or some combination of these)? If not through a website, which entity(ies) will be conducting the various activities? If different entities will conduct different parts of the data collection, be clear about which entity will conduct what part. Will that entity receive and/or maintain any personal identifiers as part of this process?

EightSeven short paper and a demographic questionnaire will be completed by both participants and non-participants. The MBSC participants will also complete a class evaluation.

MBSC class participants will complete the all eight questionnaires prior to participation in the class 1, after class 5r they have completed the series, and at a two-month follow up. Therefore, besides the demographic questionnaire, they will complete a total of 24 1 questionnaires in three time points (3 sets of 87 questionnaires at each time point). In addition, before classes 2, 3, 4, and 5 tThey will also complete fourthree short of the questionnaires (MBI, Stress/Anxiety Thermometer (1 item), MAAS at Time of Assessment (5 items), and Mindful Self-Care (3 items) and after classes 1, 2, 3, and 4, they will complete three short of the questionnaires (Stress/Anxiety Thermometer (1 item), MAAS at Time of Assessment (5 items), and Mindful Self-Care (3 items))before and after each of the 5 class sessions. Therefore tefore, they will complete a total of 2830 questionnaires during the program (10 sets of 3 questionnaires) in addition to the above three time points25 questionnaires. The MBSC participants will also complete a class evaluation.
The non-MBSC class participants will receive the same eight questionnaires in three times points, i.e. before the start of the class, at the conclusion of the class, and at a follow up as appropriateas the MBSC class but with slightly different frequency.
They will complete the questionnaires before, after the classes and at a two-month follow up. Therefore, they will also complete a total of 21 questionnaires in these three time points (3 sets of 7 questionnaires at each time point). They will be also asked to fill out an activity record during the same 1.5 hours that the MBSC participants were in class, and the same 3 questionnaires as the MBSC group for a total of 15 questionnaires (5 sets of 3 questionnaires). The non-MBSC class participants will pick up paper copies of the questionnaires from the instructor and will receive unique numbers following the same process above.
Each participant will be provided with a binder that includes uniquely numbered questionnaires with appropriate weekly designation. The non-MBSC class participants will pick up paper copies of the questionnaires from the instructor and will receive unique numbers. The instructor will not maintain any link between these numbers and the participants names or other identifiers. The numbered questionnaires will allow the instructor to link the questionnaire data to the same participant without the use of any personal identifiers. No personal identifiers will be maintained that can be linked to the data. For participants, aAttendance will be obtained via an attendance sheet that will include numbers only.
Dr. Ameli will oversee the data collection. The completed questionnaires by the MBSC group will be collected after each class. The packet of numbered questionnaires will be handed to non-MBSC group in numbered envelopes in person and collected in person at the designated time pointse conclusion of the program. Group email reminders will be sent out by the instructor to remind both groups to fill out the designated questionnaires. Both groups will be reminded that they do should not send in this information by email.

Only answer question #6 if contact information for the NIH staff will be provided to subjects in case they have questions about the research OR if NIH staff will be interacting with subjects for this project; for example:
a. to conduct screening or consent;
b. to invite them to participate in the research;
c. to email them a web link to access a survey;
d. to mail a paper copy of a survey; or
e. to collect data via interview/focus group, etc.

When contact information for NIH staff is being provided, or NIH staff are interacting with potential subjects, you must provide information to subjects that includes certain elements of informed consent for research and upload it in the system. (See Required Elements of Consent Language below.)
6. How will consent information be shared (e.g. mailed or emailed to potential subjects before data collection, read aloud to participants over the telephone or in person, or included as part of an on-line instrument, etc.)? Whose contact information will be provided to subjects in case they have questions about the research? (This person should be one of the researchers involved in the project.) If applicable, which entity, will read the consent language aloud? Will the subject sign a hard copy informed consent form (ICF) or provide electronic consent? (Note: Obtaining written or electronic consent is not mandatory for exempt research.) If a signed ICF will be obtained, who will receive the signed forms?
Consent information will be provided to the class participants both in writing and verbally and to non-participants in writing. The initial email to the NIH staff and trainees fellows interested in the MBSC program will include the following information about the evaluation:
We will be conducting an evaluation As part of the implementation of the Mindfulness Based Self-Care (MBSC) program, we will be conducting an evaluation to look at the programs effectiveness in reducing stress and enhancing wellbeing and self-care among the NIH staff and trainees. clinical fellows. In addition to assessment of the programs effectiveness, Tthe evaluation is being conducted for research purposes. We will ask you to complete a demographic questionnaire and eightseven short self-administered pre- and post-questionnaires at three time points for a total of 21 questionnaires. Before the first class, at the conclusion of the 5 classes, and at a two-month follow up. This should take 15 minutes or less each time. We will also ask you to complete four three similar shortof the questionnaires in the beginning and three at the end of each class for a total of 30 questionnaires. This should take less than 5 minutes each time. The questions will be focused on mindfulness, positive and negative mood states, self-care, and your general level of stress/distress. Your participation in the evaluation is completely voluntary., i.e. you can choose to participate in the program without participating in the evaluation . Your responses will be collected anonymously. Please call or email Rezvan Ameli, Ph.D. (Rezvan.ameli@NIH.Gov; tel: 301 402 7360) with any questions or concerns about this activity.
The initial email to the non-MBSC class participants will include the following information:
We will be conducting an evaluation of the As part of the implementation of the Mindfulness Based Self-Care (MBSC) program, we will be conducting an evaluation to look at the methods that the NIG staff and trainees clinical fellows use to reduce their stress and enhance their wellbeing and self-care. The evaluation is being conducted for research purposes. We are asking you to complete a demographic questionnaire and eightseven short self-administered questionnaires at three time points for a total of 21 questionnaires. This should take about 15 minutes or less each time. The questions will be focused on mindfulness, positive and negative mood states, and your general level of stress/distress. We will also ask you to keep a record of your activity/ies and fill out three brief questionnaires during five specified time points and fill out three brief questionnaires for a total of 15 questionnaires (less than 5 minutes each time). Your participation is completely voluntary. Your responses will be collected anonymously. Please call or email Rezvan Ameli, Ph.D. (Rezvan.ameli@NIH.Gov; tel: 301 402 7360) with any questions or concerns about this activity.

7. Which entity(ies) will have access to the individual subjects responses? Will data or results be shared between entities? Who will share data and what specifically will be shared (e.g. data and/or results; if results, aggregate or individual level)? Will it include identifiers? If so, what type of identifiers will be included? If NIH will have access to identifiable data as a part of this project, describe how you will maintain the confidentiality of the data, e.g. Confidentiality of data is ensured by good data practices, including, as applicable, locked file cabinets, storage of electronic data on the network that has firewall protection, strong passwords on computer files, and data access only for those involved in the study. (NIH HRPP SOP 6, Section 6.12). Which entity(ies) will be responsible for analysis of data?

Will the results be published or presented in conferences, etc.? Which entity(ies) will be involved in authoring these documents?

Dr. Ameli will receive the completed questionnaires and will be responsible for the data management and analysis. She will share a summary of results with Ann Berger, M.D. Chief of Pain and Palliative Care Service. Dr. Berger will share this information with appropriate NIH supervisors training directors at her discretion. Publications or conference presentations will also be decided under Dr. Bergers discretion with Dr. Amelis input.

8. If any collaborators or sub-contractors are involved, do they have IRB approval or an exemption from IRB review for their role in the project? If not, is this in process? Please note, IRB approval may be required, if we determine these individuals to be engaged in human subjects research as part of this project.

No non-NIH staff will be involved in the project.evaluation of this program.

Required Elements of Consent Language

If your research project must include consent language, according to question #6 above, the language should include the following elements: 1) that the activity is being conducted for research purposes; 2) that participation is voluntary; 3) a description of the procedures involved (e.g. approximate time commitment, number of questions, number of follow ups, etc.), and 4) the name and contact information for one of the researchers. Your project will not be approved unless each of these elements is included as part of the instrument/interview script, recruitment script/email, or as a separate consent document uploaded into the system.

In addition, you should consider addressing the following topics: 1) purpose of the research; 2) description of the subjects being targeted (number and eligibility criteria); 3) any anticipated risks or benefits; 4) methods for withdrawal from the study and whether subjects data will be maintained after withdrawal; 5) whether personal identifiers will be collected or not; 6) if identifiers will be collected, how confidentiality will be ensured (see examples in question #7 above); and 7) compensation.

SECTION V - RESEARCH INVOLVING EDUCATIONAL RESEARCH OR TESTING, SURVEY OR INTERVIEW PROCEDURES, OR OBSERVATION OF PUBLIC BEHAVIOR

Question
"Specify the types of research or procedures involved in this project: (Select all that apply)"

Your Answer
- Survey

Question
"Other - please enter additional information here"

Your Answer
** DID NOT ANSWER **

Question
"How will data collection be conducted? (Select all that apply)"

Your Answer
- In-person at an NIH site
- Other, specify below:

Question
"In-person at my collaborator's institution(s) or research site(s) - please enter additional information here"

Your Answer
** DID NOT ANSWER **

Question
"In-person at another site(s) - please enter additional information here"

Your Answer
** DID NOT ANSWER **

Question
"Other - please enter additional information here"

Your Answer
Possibly completed by participants in their homes

Question
"Who will be conducting the data collection? (Select all that apply)"

Your Answer
- Other, specify below:

Question
"Off-site contractor - please enter additional information here"

Your Answer

** DID NOT ANSWER **

Question
"Online survey tool - please enter additional information here"

Your Answer
** DID NOT ANSWER **

Question
"Other - please enter additional information here"

Your Answer
Participants will complete the surveys on paper

Question
"What is the age range of subjects involved in the research?"

Your Answer
- Adults aged >= 18 years

Question
"* If children aged less than 18 years and the project involves observation of public behavior, will the NIH investigator(s) participate in the activities being observed?"

Your Answer
** DID NOT ANSWER **

Question
"Does your project fall into any of the categories of 'clinical research' as defined by the NIH? (See NIH Glossary & Acronym List for the full NIH definition of 'clinical research'.)"

Your Answer
- NONE OF THE ABOVE

Question
"If the Planned Enrollment Report is required per above, confirm that it has been approved by the IC approver as scientifically valid: (This step must be completed before uploading)"

Your Answer
** DID NOT ANSWER **

Question
"What is the z-number for this project?"

Your Answer
** DID NOT ANSWER **

Question
"Upload Planned Enrollment Report"

Your Answer

** DID NOT ANSWER **

Question
"Upload Consent Language/Script"

Your Answer
** DID NOT ANSWER **

Question
"Upload Survey Instrument /Interview Questions /Focus Group Script"

Your Answer
Combined_measures_9_13_17.docx

Question
"Additional Info:"

Your Answer
The consent information is included in the narrative template. Here is the tracked version of combined measures:
Demographic information and course evaluation

Demographic Questionnaire
1. How old are you?

2. What is your gender? Male Female Other (describe)___________________________

3. What is your race?
American Indian/Alaska Native Asian Black or African American Caucasian Native Hawaiian/Pacific Islander Mixed/Two or more (describe) ___________________________
Other (describe) ___________________________

4. Are you Hispanic or Latino? Yes No

5. What is your marital status?
Single Married Divorced/Separated
Widowed Living with partner Other (describe)__________________
6. What is the highest level of education you have completed?
Completed grade school   Completed high school/GED
Completed vocational training Some college/university
Completed college/university Completed graduate school/advanced degree
Other (describe)___________________________
7. What is your religion?
Christianity Islam Hinduism Buddhism Judaism Agnostic
Atheist Not affiliated Other (describe) ___________________________

8. What is your employment status?   Full time Part time Not employed
Other (describe) ___________________________

9. If working, what kind of work do you do?
10. Please rate your current level of stress:
No stress

Mild
Moderate
Severe
Extreme
Other (describe)____________________________

11. Please list 3 main sources of stress:
1.
2.
3.
Others (please list):

12. What is your current level of social support (friends, family, community, religion/spirituality, other)?
No support
Some support
Good support
Excellent support
Other (describe) ____________________________

13. Please list the 3 main sources of support:
1.
2.
3.
Other (describe)____________________________

14. Do you have a medical diagnosis? Yes No

If yes, please list:
1.
2.
3.
15. How do you rate your current overall health status?
Poor
Manageable
Satisfactory/Fair
Good
Excellent
Other (describe)____________________________

16. Do you have a psychiatric diagnosis? Yes No
If yes, please list:
1.
2.
3.

17. How do you rate your current overall mental health status?

Poor
Manageable
Satisfactory/Fair
Good

Excellent
Other (describe)___________________________

18. How do you rate your quality of life?

Poor
Manageable
Satisfactory/Fair
Good
Excellent
Other (describe) ___________________________
MBSC Course Evaluation
Your evaluation will be greatly appreciated and will inform our self-care program for our staff and trainees. Thank you in advance.

Not 1 2 3 4 5 Very at all Much
How effective was the course for you in the following ways:

Help reduce your overall stress level at work? 1 2 3 4 5

Help reduce your overall stress in your personal life? 1 2 3 4 5

Help you practice mindfulness formally or informally? 1 2 3 4 5

Motivated you to take better care of yourself? 1 2 3 4 5

Help improve general quality of life? 1 2 3 4 5

How many sessions did you attend (please circle one)?

1 2 3 4 5

Rate the following practices that were introduced in class, based on your preference from 1-15. The practice you preferred most will be assigned 1, the one you preferred next would be 2 etc. You can use the same rating for more than one practice if two or more practices were equally preferred. (E.g. several practices could be designated as 1 or 2 etc. The larger numbers will indicate your least preferred practice/s.)

Practice # Practice
Mindful Breathing Mindful seeing and contemplation
Body Scan Four Rivers of Life
Mindful Eating Smiling
Mindful Walking Gratitude
Mindful Movements List of Joys
Sound as an anchor Mindful Pause
Loving Kindness meditation Tips for Mindfulness at work
R.A.I.N. practice

How do you rate the overall quality of this course?

(Poor) 1__   2__   3__   4___   5__ (excellent)

How do you rate the material in the binder that was prepared for you?

(Poor) 1__   2__   3__   4___   5__ (excellent)

Do you have any suggestions or comments that could make future Mindfulness classes more helpful?

Do you have suggestions for the teacher?

Do you have any other suggestions?

Complete set of measures administresedadministered at 3 times points to both participants and non-participants
Please indicate your level of Stress/Anxiety during the past week including now:

MASS: Experiences at Time of Assessment

Instructions: Using the 0-6 scale shown, please indicate to what degree you have each experience described below. Please answer according to what really reflects your experience rather than what you think your experience should be.

not at all   very much
1. I was finding it difficult to stay focused on what was happening.   0 1 2 3 4 5 6

2. I was doing something without paying attention.   0 1 2 3 4 5 6

3. I was preoccupied with the future or the past.   0 1 2 3 4 5 6

4. I was doing something automatically, without being aware of what I was doing.   0 1 2 3 4 5 6

5. I was rushing through something without being really attentive to it.   0 1 2 3 4 5 6

Mindful Self-Care

Check the box that reflects the frequency of your behavior (how much or how often) within past week (7 days) including today:

Never zero days (0) Rarely one day (1) Sometimes-2 to 3 days (2) Often-3 to 5 days (3) Regularly-6 to 7 days (4)

1. I engaged in a variety of self-care strategies (e.g., mindfulness, support, exercise, nutrition, spiritual practice).

2. I planned my self-care
3. I explored new ways to bring self-care into my life

Perceived Stress Scale- 10 Item

Instructions: The questions in this scale ask you about your feelings and thoughts during the last month. In each case, please indicate with a check how often you felt or thought a certain way.

1. In the last month, how often have you been upset because of something that happened unexpectedly?
___0=never ___1=almost never ___2=sometimes ___3=fairly often ___4=very often
2. In the last month, how often have you felt that you were unable to control the important things in your life?
___0=never ___1=almost never ___2=sometimes ___3=fairly often ___4=very often
3. In the last month, how often have you felt nervous and "stressed"?
___0=never ___1=almost never ___2=sometimes ___3=fairly often ___4=very often
4. In the last month, how often have you felt confident about your ability to handle your personal problems?
___0=never ___1=almost never ___2=sometimes ___3=fairly often ___4=very often
5. In the last month, how often have you felt that things were going your way?
___0=never ___1=almost never ___2=sometimes ___3=fairly often ___4=very often
6. In the last month, how often have you found that you could not cope with all the things that you had to do?
___0=never ___1=almost never ___2=sometimes ___3=fairly often ___4=very often
7. In the last month, how often have you been able to control irritations in your life?
___0=never ___1=almost never ___2=sometimes ___3=fairly often ___4=very often
8. In the last month, how often have you felt that you were on top of things?
___0=never ___1=almost never ___2=sometimes ___3=fairly often ___4=very often
9. In the last month, how often have you been angered because of things that were outside of your control?
___0=never ___1=almost never ___2=sometimes ___3=fairly often ___4=very often
10. In the last month, how often have you felt difficulties were piling up so high that you could not overcome them?
___0=never ___1=almost never ___2=sometimes ___3=fairly often ___4=very often

Cohen, S., Kamarck, T., Mermelstein, R. (1983). A global measure of perceived stress. Journal of Health and Social Behavior, 24, 385-396.

Geneva Emotion Wheel Version 3.0

Instructions

This instrument, called the Geneva Emotion Wheel, is used to measure as precisely as possible the emotions you experienced during the past week including today and right now.

Our emotions are often blended or mixed and contain many different components. Please rate the intensity of all the emotions in the wheel with respect to what you felt, even if the intensities are very low. For those emotions that were not at all part of your reaction, please check the small box under the smallest circle, respectively.

The PANAS

This scale consists of a number of words that describe different feelings and emotions. Read each item and then mark the appropriate box. Indicate to what extent you have experienced these feelings during the past week including today and right now. Use the following scale to record your answers.

1

Very slightly or not at all 2

A little 3

Moderately 4

Quite a bit 5

Extremely

Interested

distressed

excited

upset

strong

Guilty

Scared

Hostile

Enthusiastic

Proud

Irritable

Alert

Ashamed

Inspired

Nervous

Determined

Attentive

Jittery

Active

Afraid

Below is a collection of statements about your everyday experience. Using the 1-6 scale below, please indicate how frequently you currently have each experience.

Please answer according to what really reflects your experience rather than what you think your experience should be. Treat each item separately from every other item.

MBI- 2-item
(Adapted from West et al, 2012, JGIM)

Never A few times a year or less Once a month or less A few times a month Once a week A few times a week Every day
How often I feel burned out from work?

How often Ive become callus toward people since you started your job?

West et al (2012) concurrent validity of single-item measures of emotional exhaustion and depersonalization in burnout assessment, J Gen Intern Med. 2012 Nov; 27(11): 14451452.Published online 2012 Feb 24. doi: 10.1007/s11606-012-2015-7

Measures per class session

Please indicate your level of Stress/AnxityAnxiety during the past week including now:

MBI- 2-item
(Adapted from West et al, 2012, JGIM)

Never A few times a year or less Once a month or less A few times a month Once a week A few times a week Every day
How often I feel burned out from work?

How often Ive become callus toward people since you started your job?

West et al (2012) concurrent validity of single-item measures of emotional exhaustion and depersonalization in burnout assessment, J Gen Intern Med. 2012 Nov; 27(11): 14451452.Published online 2012 Feb 24. doi: 10.1007/s11606-012-2015-7

MASS: Experiences at Time of Assessment

Instructions: Using the 0-6 scale shown, please indicate to what degree you have each experience described below. Please answer according to what really reflects your experience rather than what you think your experience should be.

not at all very much

1. I was finding it difficult to stay focused on what was happening. 0 1 2 3 4 5 6

2. I was doing something without paying attention. 0 1 2 3 4 5 6

3. I was preoccupied with the future or the past. 0 1 2 3 4 5 6

4. I was doing something automatically, without being aware of what I was doing. 0 1 2 3 4 5 6

5. I was rushing through something without being really attentive to it. 0 1 2 3 4 5 6

Mindful Self-Care

Check the box that reflects the frequency of your behavior (how much or how often) within past week (7 days) including today:

Never zero days (0) Rarely one day (1) Sometimes-2 to 3 days (2) Often-3 to 5 days (3) Regularly-6 to 7 days (4)

4. I engaged in a variety of self-care strategies (e.g., mindfulness, support, exercise, nutrition, spiritual practice).

5. I planned my self-care

6. I explored new ways to bring self-care into my life

17-NIMH-00118: "Mindfulness Based Self Care (MBSC)" STUDY STATISTICS

Sample Size

Using PASS software, sample size was determined based on a "Test for Two Means in a Repeated Measures Design." This compared MBSC and control groups, from baseline to post-intervention. Using the primary outcome of PSS-10, a detection of a 1.0 difference was determined to be a meaningful change.

Sample size calculation details included: two-sided test, power set at 0.90, alpha at 0.05, with equal group allocation. The effect size tested a range of differences from 0.25 to 1.5 in increments of 0.25. Since the primary comparison was between MBSC and controls from baseline to post-intervention, repeated measurements were specified as two intervals.

Due to the repeated measure, the covariance structure specifications were set as compound symmetry, with a sigma of 1, and rho of 0.5.

The estimates yielded the following range of sample sizes:

Numeric Results

Two-Sided Test. Null Hypothesis: D = 0. Alternative Hypothesis: D ≠ 0.
Covariance Type = Compound Symmetry

Target Power	Actual Power	N1	N2	N	M	D1	Sigma	Rho	Alpha
0.90	0.90092	253	253	506	2	0.250	1.000	0.500	0.050
0.90	0.90423	64	64	128	2	0.500	1.000	0.500	0.050
0.90	0.90951	29	29	58	2	0.750	1.000	0.500	0.050
0.90	0.90423	16	16	32	2	1.000	1.000	0.500	0.050
0.90	0.92293	11	11	22	2	1.250	1.000	0.500	0.050
0.90	0.93373	8	8	16	2	1.500	1.000	0.500	0.050

For the detection of a score of 1.0, a total of 32 subjects would be needed (16 per group). A drop-out rate of 10-15% would require an additional 1-2 subjects per group, for a total of 34-36 subjects.
To detect a smaller difference (ie, 0.750), a total of 58 subjects (29 per group) would be needed.

For a power of 0.80, the following estimates were calculated:

Numeric Results

Two-Sided Test. Null Hypothesis: D = 0. Alternative Hypothesis: D ≠ 0.
Covariance Type = Compound Symmetry

Target Power	Actual Power	N1	N2	N	M	D1	Sigma	Rho	Alpha
0.80	0.80130	189	189	378	2	0.250	1.000	0.500	0.050
0.80	0.80743	48	48	96	2	0.500	1.000	0.500	0.050
0.80	0.80130	21	21	42	2	0.750	1.000	0.500	0.050
0.80	0.80743	12	12	24	2	1.000	1.000	0.500	0.050
0.80	0.82298	8	8	16	2	1.250	1.000	0.500	0.050
0.80	0.85084	6	6	12	2	1.500	1.000	0.500	0.050

The MBSC training with a 15-person class size would need to be repeated three times in order to ensure adequate number of participants in each group, accounting for drop-outs and missing data.

Statistical Analysis Plan

The study is a randomized control trial of MBSC intervention vs controls. The sessions will be repeated three times, enrolling 15 participants and 15 controls each time to ensure adequate numbers.

The analysis will be intent-to-treat based on randomized group allocation.

The primary outcome is PSS-10, compared between MBSC and controls, and within groups over time. The secondary outcomes are VAS-A, MBI-2, MAAS-T, MAAS-S, MSCS-G, and PANAS-pos, and PANAS-neg. These will be obtained at three time points for the MBSC group (baseline, post-intervention, and 13-week follow-up), and twice for the control group (baseline and post-intervention).

Demographic characteristics include age, sex, race, ethnicity, marital status, religion, current position, presence of medical conditions, and presence of psychiatric conditions.

Data will be assessed for their distributional assumptions, and analyzed accordingly either with parametric or non-parametric methods, as appropriate. Continuous data between MBSC and controls will be compared using two-sample t-tests or Wilcoxon rank sum tests. Categorical data between groups will be compared using chi-square or Fisher's exact test, as appropriate.

For the main analyses, generalized linear mixed modeling (PROC MIXED) for repeated measures will be used to compare post-intervention and follow-up measures for each outcome. These models will involve both between and within group analyses, utilizing statistical power and accounting for missing data. In addition, within-subject correlations will be accounted for by compound symmetry covariance structure. Post-hoc comparisons will be corrected using the Bonferroni method.

Effect size will also be computed for both between and within groups, using bias-corrected Cohen's d (Hedges' g).

Data will be described by frequency (percentages), mean (SD), median (IQR), and 95% Confidence Intervals, accordingly.

Data will be analyzed using SAS v9.4 (SAS Institute, Inc, Cary, NC).
