# Supplementary material for: Effect of a Brief Mindfulness-Based Program on Stress in Health Care Professionals at a US Biomedical Research Hospital: A Randomized Clinical Trial
Source: JAMA Netw Open. 2020 Aug 25;3(8):e2013424. doi: 10.1001/jamanetworkopen.2020.13424 (PMC7448827; doi:10.1001/jamanetworkopen.2020.13424)
Supplement: Supplement 2. — Data Sharing Statement [file jamanetwopen-3-e2013424-s002.pdf]

Data Sharing Statement

Ameli. Effect of a Brief Mindfulness-Based Program on Stress in Health Care Professionals at a US Biomedical Research Hospital. *JAMA Netw Open*. Published August 25, 2020.
10.1001/jamanetworkopen.2020.13424

Data
Data available: No
